# Correction: Examining trip-level errors in passively collected mobile device data for data quality assurance

**DOI:** 10.1371/journal.pone.0326156

**Published:** 2025-06-10

**Authors:** The PLOS One Staff

## Notice of Republication

This article was republished on May 23, 2025, to correct error in the reference 18 that was introduced during the typesetting process. The publisher apologizes for this error. Please download the article again to view the corrected version. Both the originally published, uncorrected article and the republished, corrected article are provided here for reference.

## Supporting information

S1 File Originally published, uncorrected article. (PDF)

S2 File Republished, corrected article. (PDF)
