# Supplementary material for: Correction: Examining trip-level errors in passively collected mobile device data for data quality assurance
Source: PLoS One. 2025 Jun 10;20(6):e0326156. doi: 10.1371/journal.pone.0326156 (PMC12151412; doi:10.1371/journal.pone.0326156)
Supplement: S1 File — (PDF) [file pone.0326156.s001.pdf]

PLOS One

RESEARCH ARTICLE

Examining trip-level errors in passively collected mobile device data for data quality assurance

Peiqi Zhang[1]*, **Kathleen Stewart**[1], **Aref Darzi**[2]

1 Department of Geographical Sciences, University of Maryland, College Park, Maryland, United States of America, **2** Center for Advanced Transportation Technology Laboratory, Department of Civil and Environmental Engineering, University of Maryland, College Park, Maryland, United States of America

* pzhang13@umd.edu

OPEN ACCESS

Citation: Zhang P, Stewart K, Darzi A (2025) Examining trip-level errors in passively collected mobile device data for data quality assurance. PLoS One 20(4): e0321970. https://doi.org/10.1371/journal.pone.0321970

Editor: Tri-Hai Nguyen, Van Lang University: Truong Dai hoc Van Lang, VIET NAM

Received: September 23, 2024

Accepted: March 13, 2025

Published: April 25, 2025

Copyright: © 2025 Zhang et al. This is an open access article distributed under the terms of the Creative Commons Attribution License, which permits unrestricted use, distribution, and reproduction in any medium, provided the original author and source are credited.

Data availability statement: **TO PA AT ACCEPT: Ensure publication of Harvard Dataverse repository.** The authors confirm that the data supporting the conclusions are described in the paper. The raw LBS data is not publicly accessible because the raw data are controlled by third-party vendors and were provided to us under a legally binding data use

Abstract

Location-based service (LBS) data passively collected by mobile devices has been widely adopted in multiple fields for its advantages in revealing travel behaviors. Data quality assessments have always been important steps for analyses using the data, but the impact of trip-level errors has not been a focus of these assessments. We examine a newly emerged type of error present at trip-level in LBS datasets that violates the spatio-temporal consistency of such data by including trips on road segments where and when there should be no trips. We designed a distributed-computing workflow to quantify the errors by comparing the number of trips on closed road segments during road closures with time periods before and after. Using two real-world cases from 2023, we examined multiple datasets acquired from major vendors in the US, and several of the datasets contained a significant number of trip-level errors. These findings point to the errors being present in recent datasets that have not otherwise been processed for data quality and can significantly impact analyses by data users. Data users should consider conducting trip-level error data quality checks as part of their preprocessing steps.

Introduction

In recent years, with the development of information technology and the increasing adoption of smart devices, location-based service (LBS) data, i.e., data that are passively collected in mobile devices by location-aware sensors such as GPS, WIFI, and Bluetooth sensors, has become widely available [1,2]. Compared with traditional actively collected data types such as survey and census data, LBS data has the advantage of larger data volumes, broad spatio-temporal coverages, and higher population coverage [3]. With these advantages, LBS data has been widely used in multiple fields including, for example, transportation science, geospatial information science, and public health for travel behavior analysis [4,5], transportation planning [6,7], transportation system management and operations [8], and pandemic-related analytics [9–11].

agreement. This agreement explicitly prohibits the dissemination of the data to external parties. These restrictions are designed to protect proprietary information and the intellectual property rights of the data providers. Please contact nhts@umd.edu for further information on the raw LBS data. However, two aggregated datasets (one for each road closure case study) and scripts introduced in this research are available at (https://dataverse.harvard.edu/privateurl.xhtml?token=62c90d96-ddd4-4116-8534-43e1029190c9). These aggregated datasets show the number of waypoints from the test datasets that were matched to the road segments for each day before, during, and after the road closures.

Funding: This research is partially supported by a U.S. Department of Transportation Federal Highway Administration (U.S.DOT FHWA) project entitled "Next-Gen National Household Travel Survey – National Origin-Destination Data Component". The opinions in this paper do not necessarily reflect the official views of U.S.DOT FHWA. The funders had no role in study design, data collection and analysis, decision to publish, or preparation of the manuscript.

Competing interests: The authors have declared that no competing interests exist.

Despite the advantages that LBS data brings, concerns about data quality have always existed because LBS data was not designed for travel behavior or transportation analysis purposes in the first place as is survey data, and ground truth data is largely unavailable [2]. For example, at waypoint level, GPS sampling errors (generated by the GPS sensors in mobile devices at 7–13 meters horizontal error on average) can impact the estimation of distances or trajectories similarity commonly used in travel behavior analyses [12–14]. To address data quality concerns, multi-dimensional LBS data assessments have been proposed and discussed, for example, assessments of accuracy and consistency [8,15]. The accuracy dimension mainly refers to spatial accuracy since temporal accuracy in LBS data is seldomly reported and hard to assess. Spatial accuracy is usually measured as the distance between waypoints' reported locations and their actual locations with a certain confidence level [8,16]. Waypoints (i.e., sightings of devices with location and other information provided at certain timestamps) with lower spatial accuracy are often dropped as part of data preprocessing. The consistency dimension is used to define certain semantic rules that the LBS data should obey, for example, the distance between waypoints in a trip should be within a range that corresponds to the expected travel speed for give travel modes of the device user [8]. Different data preprocessing methods have been developed in previous works to ensure the spatial or temporal consistency of LBS data such as filtering based on irregular turns and speed [17], Kalman and Particle filters [18–20], and outlier detection [21,22].

For this research, we discuss the occurrence of an unprecedented type of LBS data error that violates both spatial and temporal consistency and that could significantly impact many types of analyses using LBS data. Based on an expectation for spatial and temporal consistency, there should be no (or nearly no) trips that go through a closed road segment, and therefore, trips that are found to be present at times when road segments were closed are errors. Unlike waypoint-level errors and the corresponding filtering and preprocessing models that are commonly discussed in studies (e.g., GPS sampling errors), these trip-level errors have not been discussed in previous studies to the best of our knowledge. In this research, using scenarios that involve road closures as test cases, we have found significant numbers of trips that were erroneous in 2023 LBS datasets acquired from multiple major data vendors in the US. In this paper, we discuss our data quality workflow to detect and quantify the trip-level errors.

The first step in the data quality workflow was to determine trip trajectories and routes followed by each of the devices in the datasets in the location of a road closure, and investigate and compare the number of trips that followed different routes (e.g., trips that were on road segments leading up to and including the location of the closure during the time of the closure and trips that followed any local detours that had been planned by local department of transportation (DOT) to allow for traffic throughflow during the closure). These different trips were examined for different time periods (e.g., before the road closure, during the road closure, and after traffic has resumed). To overcome the challenges of large data volume, the workflow was designed using the distributed computing environment Apache Spark and Sedona on AWS [23–25].

For this research, we used two real-world cases for trip-level error examination: a road closure on Interstate 95 (I-95) in Philadelphia, PA, that began on June 11th, 2023, and a road closure on the Interstate 10 (I-10) in Los Angeles, CA, that began on November 11th, 2023. For the road closure in Philadelphia, we examined trip-level errors in 4 LBS datasets acquired from 4 major data vendors for different time periods. In addition, we also compared the pattern of different number of trips for different routes showed by the datasets. For the second case in Los Angeles, we examined 5 LBS datasets acquired from 5 major data vendors also for three time periods and compared the heterogenous pattern of trips showed among the datasets.

Although the trip-level errors were found in scenarios involving road closures, we believe the LBS datasets containing these trip-level errors are suspect and may include additional trip-level errors elsewhere. We first noticed this data quality concern during our quality assurance/quality control processes conducted on 2023 LBS data. Further investigation would be necessary to better understand the extent of these errors in the LBS data market, both spatially and temporally.

Methods

Study areas and datasets

We analyzed two cases involving road closures caused by serious accidents to examine trip-level errors, i.e., examine whether there were trips that went through the closed road segments during the time of the road closures. The first case was based on a road closure that occurred on a segment of the I-95 Interstate in northeast Philadelphia, PA, due to a serious accident and related fire under an overpass of the I-95 on June 11, 2023 (Fig. 1). This accident resulted in a roughly 9-mile closure of the I-95 until June 23, 2023, and detours were set up to redirect traffic away from the closed road segments (Pennsylvania DOT, https://www.penndot.pa.gov/RegionalOffices/district-6/Pages/AlertDetails.aspx) *.

For this case, we examined trips from 4 passively collected LBS datasets from 4 different data vendors (datasets A to D respectively) that covered the period before the closure (June 6th to June 10th), during the closure (June 11th to June 22nd), and after the closure (June 23rd to June 29th). All the datasets were comprised of GPS waypoints where each waypoint contained attributes including unique anonymized device ID, longitude, latitude, and timestamp. To avoid the potential impact brought by preprocessing (i.e., different types of filtering), we used raw data and did not include any commonly used preprocessing steps. The raw data may contain trip trajectories sourced from a mix of travel modes (e.g., driving, cycling, and pedestrian trips) but since all of our analyses were designed for freeways and traffic moving at freeway speeds, it is less likely there are trips in types other than driving showed in our analysis. In addition, in the trip-level data quality inspection we introduced in the later section, we further eliminate the impact of trips in types other than driving.

To better compare these datasets and to reduce the computation, we created a bounding box based on the location of the closure and local detours, and extracted only the waypoints within the bounding box from all the datasets. With respect to the number of devices and waypoints that were extracted, dataset B contains significantly fewer number of devices and waypoints compared with the three other datasets (Table 1). The 4 datasets showed a generally similar number of waypoints per device daily in the study area, with a range from 28 to 40 approximately.

The road network data used for analysis was extracted from OSM and contained approximately 6,700 road segments in the bounding box. According to the description in the road closure announcement by the PA DOT (Pennsylvania DOT, https://www.penndot.pa.gov/RegionalOffices/district-6/Pages/AlertDetails.aspx), we extracted 11 road segments as closed segments for this study (shown in red in Fig. 1)

The second case was another road closure that occurred on a segment of the I-10 in downtown Los Angeles, CA on November 11, 2023 (Fig. 2). The I-10 was closed to traffic from November 11 to November 19 due to a major fire caused by an accident nearby (Caltrans, https://dot.ca.gov/fix-10). For this case, we tested and compared 5 LBS datasets that covered the period before the road closure (November 4th to November 10th), during the closure (November 11th to November 19th), and after traffic had resumed (November 20th to November 26th) and we used a bounding box to select only the waypoints within the study area for this analysis. In this case, datasets B and E contained fewer devices and waypoints

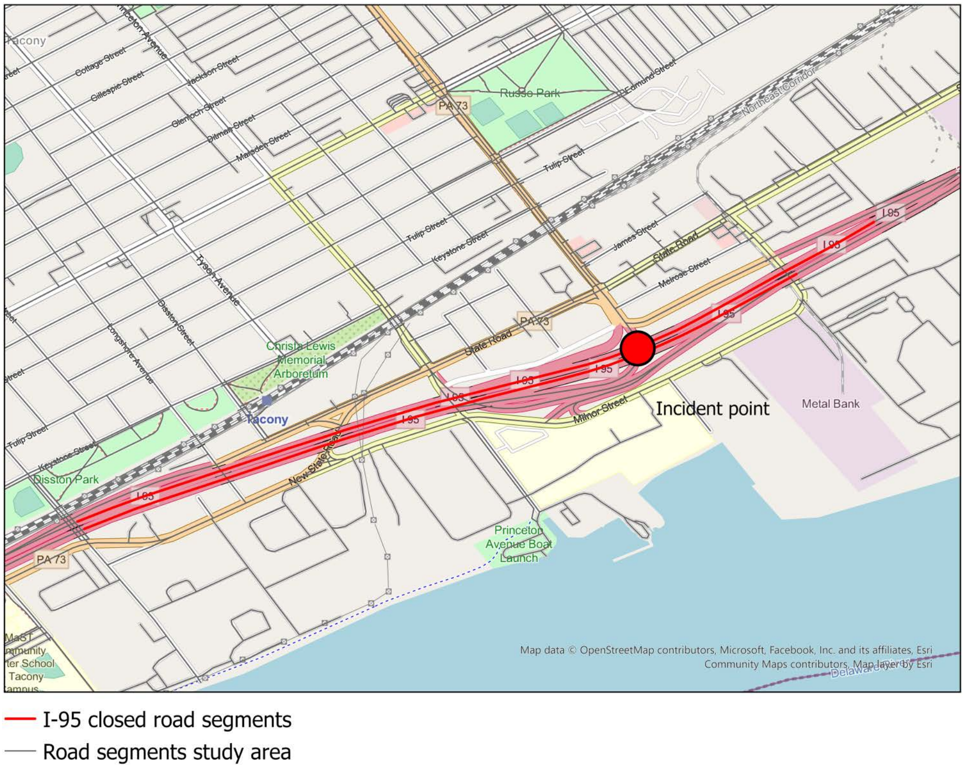

Fig 1. Incident and road closure along I-95 in Philadelphia, PA, June 11th, 2023.

https://doi.org/10.1371/journal.pone.0321970.g001

Table 1. Mean daily number of devices and waypoints for the two cases, dataset E was not used for case 1 analysis.

	Case 1 (Philadelphia, PA)		Case 2 (Los Angeles, CA)	
	**Mean daily no. of devices**	**Mean daily no. of waypoints**	**Mean daily no. of devices**	**Mean daily no. of Waypoints**
**Dataset A**	63727	1766940	72219	1177681
**Dataset B**	1731	68609	1833	105767
**Dataset C**	37669	1193366	42190	729419
**Dataset D**	121296	4569839	114694	2582876
**Dataset E**	–	–	1756	76794

https://doi.org/10.1371/journal.pone.0321970.t001

compared with the other 3 datasets (Table 1). As for number of waypoints per device, unlike case 1, datasets B and E contained more waypoints per device daily (58 and 44 respectively) than the other 3 datasets (16, 17, and 23 respectively). We also used raw data for this case study and used road network data from the Open Street Map (OSM) [26].

Map matching

To compute and compare the number of trips that went through the closed segments, the first step was to determine the trips and the routes followed by each device holder by aligning the location of each waypoint to the most appropriate road segment (i.e., a map matching process). In this research, based on waypoints that are extracted by the bounding boxes, we define a trip as a series of waypoints with the same device id and consecutive timestamps. Considering the circumstances that device users could drive through the bounding boxes multiple times during study time, we define that if the

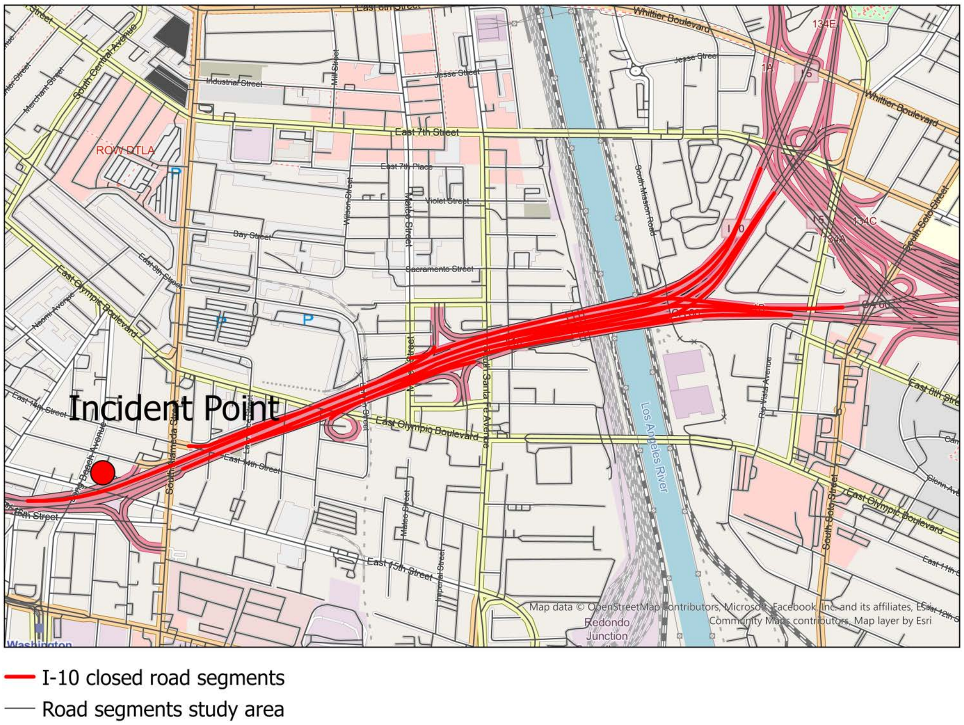

Fig 2. Incident and road closure along I-95 in Los Angeles, CA, November 4th, 2023.

https://doi.org/10.1371/journal.pone.0321970.g002

timestamp difference between two adjacent waypoints of a trip exceeds a threshold of 30 minutes, we divide it into two trips at these two waypoints. We then applied a distributed map matching module for the trips designed based on Apache Spark and Sedona [23,24,27] that was proposed in Zhang et al., (2023) [17] for the trips. Taking the advantage of the distributed computing environment, the module was designed to overcome the challenges of large data volume and broad spatial scale. Because of the GPS sampling error and the dense distribution of urban road networks, it is possible that road segment closest to the location of a recorded waypoint is not the road segment that this waypoint actually passed through [28,29]. Therefore, the map matching module considered both the distance between the waypoint and the candidate road segments and the angle between the driving direction and the road segment direction to select the most appropriate road segment for map matching [17,30].

The framework was applied on Amazon AWS EMR with 1 master node and 10 working nodes. For each working node, we used EMR C5 4Xlarge instance that contained 16 core CPUs and 32 GB memory.

Trip-level data quality inspection based on road closure scenarios

Our data quality check used scenarios involving road closure and was based on the expectation that there should be no (or nearly no, excluding potential emergency vehicle or maintenance workers) trips that occur on the closed road segments during the time of closure (segment *a* in Fig. 3a). Based on the contrapositive of this, we have the assumption that the data quality of an LBS dataset was suspect if it contained trips that went through closed road segments during a road closure event.

Due to the existence of waypoint-level errors in LBS datasets, one or several waypoints of a trajectory could show up or be matched to a road segment while a vehicle was actually traveling on a nearby route even after the map-matching module had already been applied to avoid this kind of mismatching. To avoid trip trajectories showing up on a closed road because

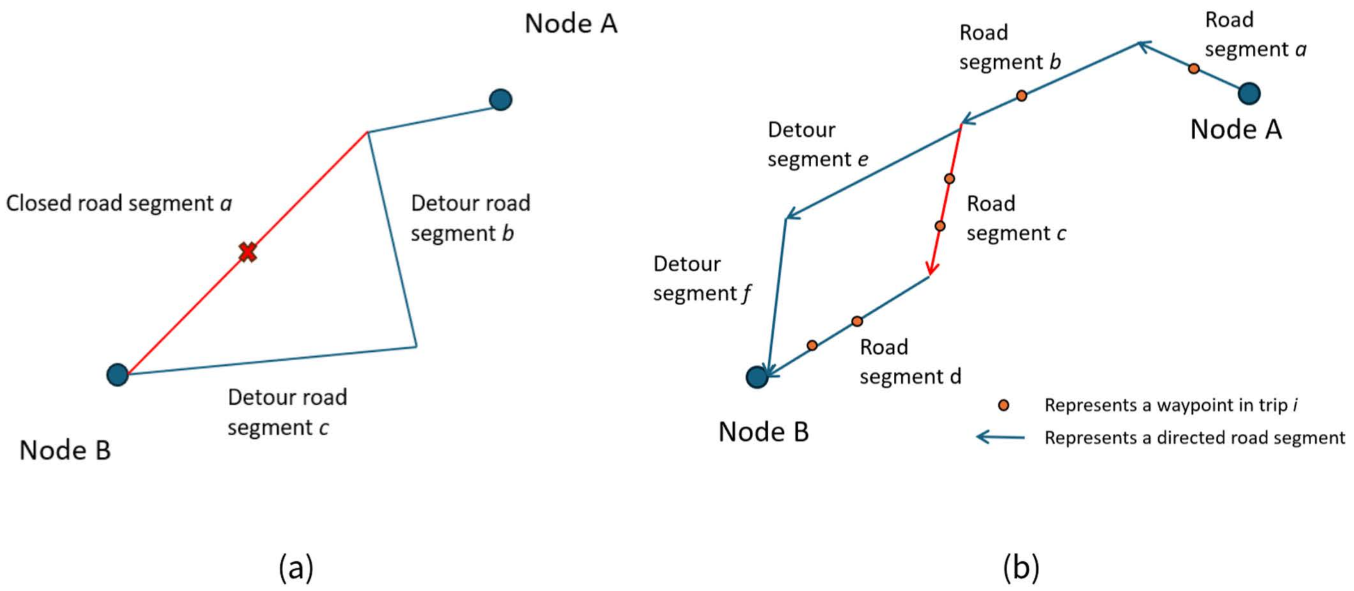

Fig 3. (a) Diagram for a road closure scenario. (b) Diagram for the trip-level analysis.

https://doi.org/10.1371/journal.pone.0321970.g003

of data noise, we developed a workflow to examine trip-level errors where we focused not only on the number of waypoints or devices that were matched to road segments (e.g., road segment *c* in Fig. 3b), but we also assumed that the same trip should have waypoints matched to the segments prior to and after those segments (e.g., road segments *a, b*, and *d* in Fig. 3b). That is, only when a trip has waypoints (two waypoints based on manual inspection of LBS data sampling intervals) that were matched not only to the closed segments, but also to the segments before and after the closure (based on the direction of travel), and the matched waypoints conform to the movement of a device along a road in a certain direction, do we consider that a trip has gone through the segments in the closure area, i.e., there were trips on closed road segments.

Based on a scenario that involves road closure (e.g., road segment c in Fig. 3b), we expect to see no trip (or nearly no trip, for potential emergency vehicles or workers) passing through the closed road segment (e.g., trips passing through road segments *a*, *b*, *c*, and *d* in Fig. 3b) but number of trips that used local detours (e.g., road segments *a, b*, *e*, and *f* in Fig. 3b) to increase during the road closure period.

Travel routes for the two cases

For the two cases in this research, we examined several travel routes for the trip-level analysis to analyze the trips that appeared to travel through the road closure area and the local detours in different time periods. For case 1, we examined 3 routes for the trip-level analysis, travel through the closed segments from east to west, from west to east, and travel on the local detours. For these routes, in addition to the closed road segments, we also extracted 24 road segments on the I-95 to the east of the closed segments (shown in blue in Fig. 4a), 12 road segments on I-95 to the west of the closed segments (shown in light blue in Fig. 4a), and the official local detour road segments to the south (shown in green in Fig. 4a). Segments to the east and west of the road closure serve as road segments before and after the closure mentioned in the previous section for all of the three routes. For travel following the local detours posted by the PA DOT, we investigated the trips that traveled through the official local detour segments from east to west to examine whether some of the original I-95 travelers used the local detours, and also use as a comparison to the number of trips through the actual road closure area.

For the routes we designed for case 1, the I-95 in the road closure area is an isolated freeway and there are no other roads (or space like parking lots) beneath this section of the I-95. Therefore, there shouldn't be any pedestrians or cycling trips returned by the trip-level analysis.

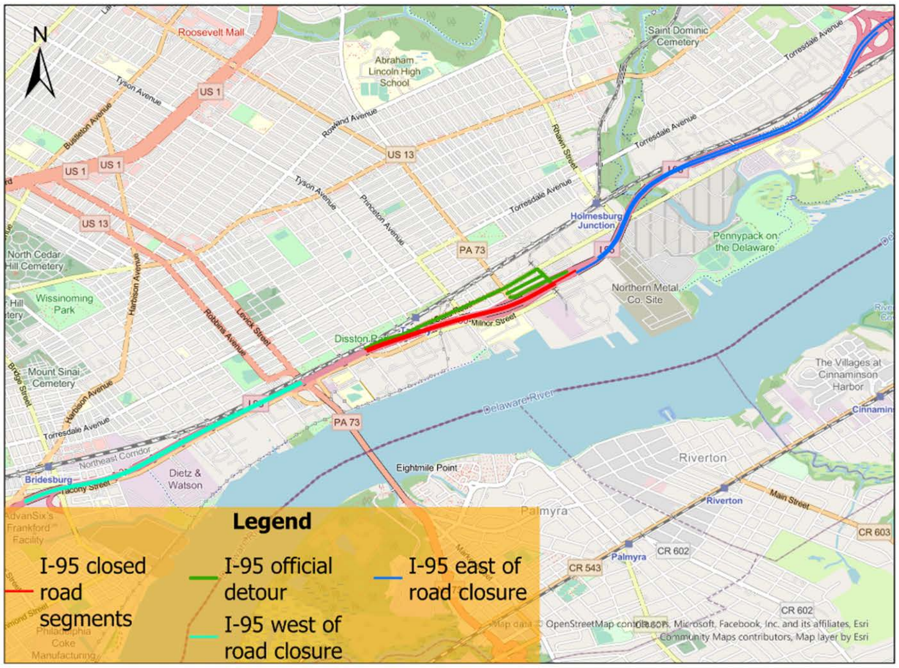

(a)

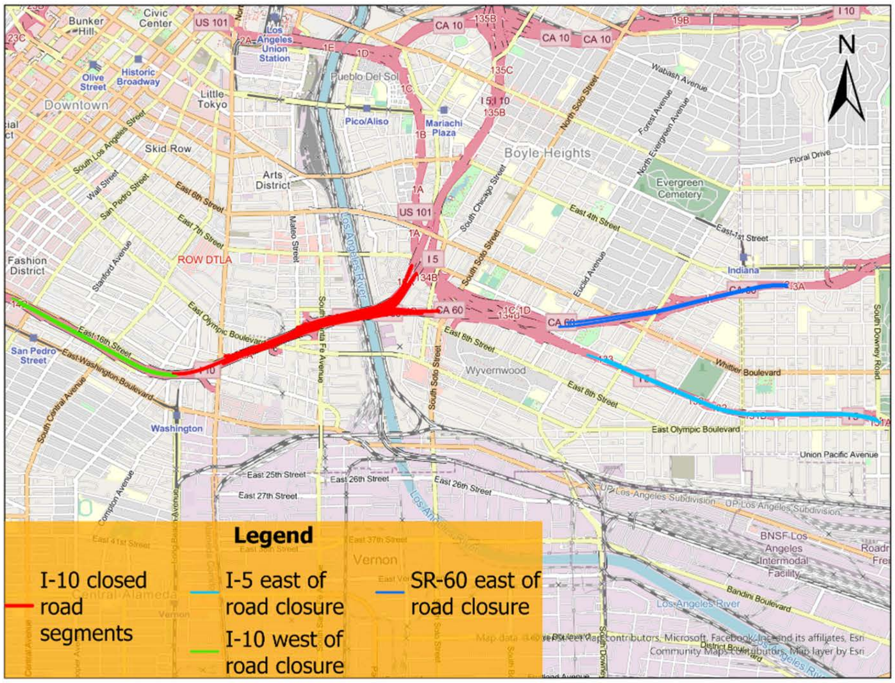

(b)

Fig 4. Travel routes used for trip-level analysis for (a) case 1, Philadelphia I-95 closure and (b) case 2, Los Angeles, I-10 closure.

https://doi.org/10.1371/journal.pone.0321970.g004

Since the road closure for case 2 was located at one of transportation hubs in downtown Los Angeles, we selected 4 routes that traveled through closed road segments and did not cover all possible routes that traveled through closed road segments. For this case, we extracted 8 road segments on I-10 to the west of the closed road segments (shown in green in Fig. 4b), 6 road segments on State Route 60 (SR-60) to the east of the road closure (shown in blue in Fig. 4b), and 23 road segments on Interstate 5 (I-5) to the east of the road closure (shown in light blue in Fig. 4b). The 4 selected routes include traveled through the road closure from I-10 to I-5 from east to west, traveled through the road closure from I-5 to I-10 from west to east, traveled through the road closure from I-10 to SR-60 from east to west, and traveled through the road closure from SR-60 to I-10 from west to east. Therefore, there shouldn't be any pedestrians or cycling trips showed in both side of the river via the freeway route we designed and returned by the trip-level analysis.

While it is possible that some segments might show DOT worker vehicles or workers' pedestrian trips on the closed segments, we assume there should be very low numbers of trips through the routes since these routes contain both closed and open road segments.

Results

Case 1, Philadelphia, PA, I-95 closure

The number of devices extracted using the bounding box of the study area was generally stable for all 4 datasets over the number of days of the closure and showed that the average number of trips for the three time periods was also shown a fluctuation within 10% (Fig. 5a). The number of waypoints extracted for the study area showed a similar pattern for datasets A and C over the three time periods (Fig. 5b). The number of waypoints from dataset D showed an increase from June 16th till 18th. The number of waypoints from dataset B also didn't show a significant fluctuation before the closure on June 11th but showed a decreased on average number of waypoints by approximately 28% for the road closure period, and kept the stable pattern for the whole incident period. The number of waypoints from dataset B bounced back to the before incident level for the period after traffic had resumed.

Devices that were matched to the road closure in Case 1

After applying map matching for all four LBS datasets, we aggregated the number of devices for the closed road segments at a daily level (Fig. 6). Before the incident, the number of devices that matched the road segments followed the

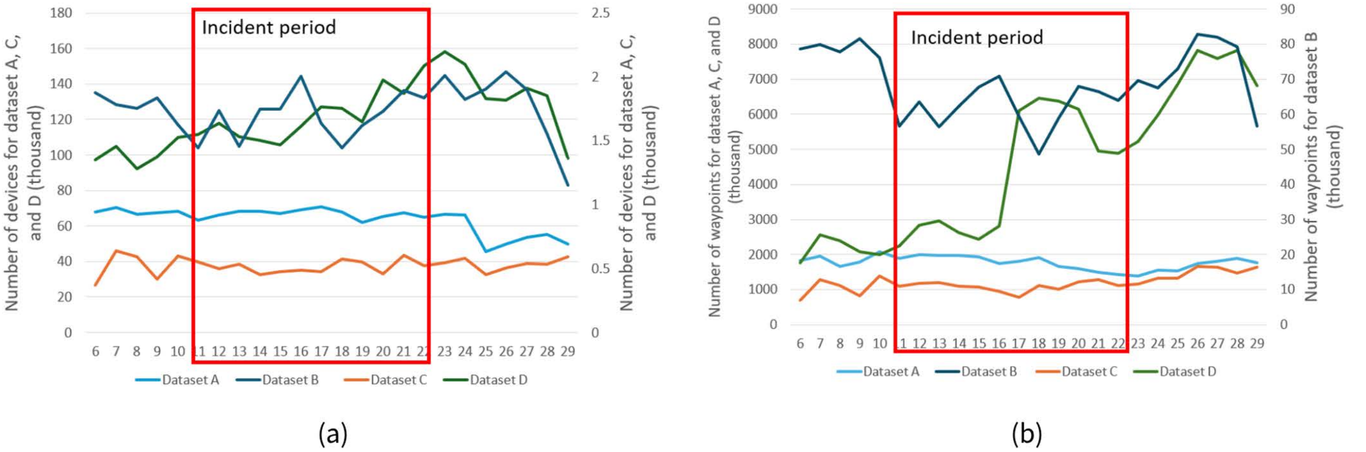

Fig 5. (a) the number devices corresponding to the 4 datasets for the study time period, (b) the number of waypoints for the datasets for the study time period. Due to dataset B containing significantly fewer waypoints and devices, we used the second y axis to represent dataset B values. The road closure period is bounded in red.

https://doi.org/10.1371/journal.pone.0321970.g005

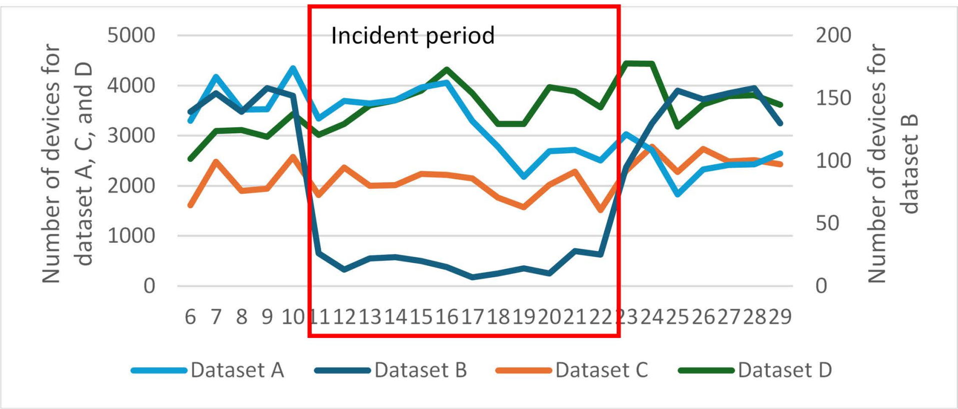

Fig 6. Number of devices that were matched to road segments on the I-95 for the study time period. The number of devices for dataset B is represented on the second axis. The road closure period was bounded in red.

https://doi.org/10.1371/journal.pone.0321970.g006

same pattern for all 4 datasets. However, the number of devices from different datasets showed completely different patterns during the road closure period. Dataset B showed a significant decrease in the daily number of devices during the road closure, with a decrease of around 88% during the road closure period compared with the average number before the incident and after the traffic had resumed. However, datasets C and D, showed a different result with the daily number of devices not changing significantly from before the incident (a fluctuation within 20% on average for the two datasets). For dataset A, the daily number of devices decreased from June 17th (during the closure period) but this decrease was less than the decrease shown by dataset B.

After the road had been re-opened and traffic had resumed, the daily number of devices matched to the closed segments showed a clear pattern of bounce back on June 23rd and June 24th and was generally stable after those dates. The daily number of devices for datasets A, C, and D followed the same trend as each other for this time period.

For before the road closure time period, both datasets A and B reasonably showed waypoints in the study area (Fig. 7 a and d), except for dataset B showed fewer waypoints than dataset A since it contained fewer waypoints in the study area (Fig. 5b). But for the road closure period, we could clearly see there were nearly no waypoints showed in the I-95 road closure area (we represented it as a buffer of the I-95 closed segments with 20 meters radius in figure) (Fig. 7e), but dataset A showed similar if not more waypoints in the road closure area (Fig. 7b). For the after the traffic resumed time period, datasets A and B showed a similar pattern as the before the road closure period (Fig. 7 c and f). Datasets C and D showed a very similar pattern as dataset A on the map for different periods so we didn't include maps for these two datasets consider the space limitation.

Trip-level analysis for case 1

The results of trip-level analysis for trips that were present on the closed road segments showed a very similar pattern for the 4 datasets to the device-level analysis results. For the period before the road closure, all datasets showed a stable pattern of go-through trips for all three of the travel routes that we analyzed.

During the road closure period, the number of trips from dataset A for the two routes (west to east and east to west) showed an approximately 30% decrease since June 16th and 17th respectively compared with the before the road closure period, which corresponded to the pattern shown by the number of devices matched to the road closure. However, there was still a significant number of trips that were on the closed road segments at times when there should be no trips. For the number of trips that used the local detours in dataset A, these trips showed a slight increase from June 12th to 17th compared with the before road closure period and then dropped to the before-period level on June 18th (Fig. 8a).

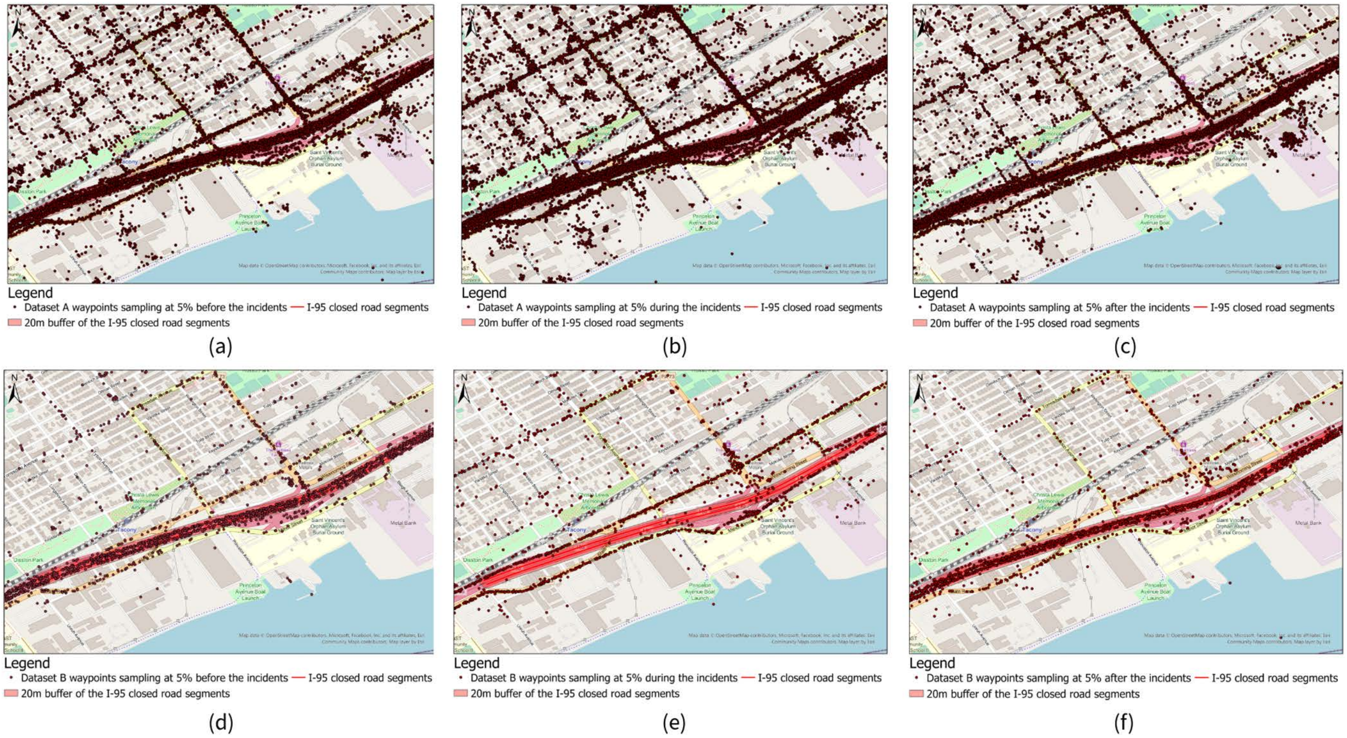

Fig 7. Waypoints randomly sampled at 5% for dataset A in I-95 road closure area of case 1 for (a) before the road closure period, (b) during the road closure period, and (c) after the traffic resumed period. And Waypoints randomly sampled at 5% for dataset B in study area for **(d)** before the road closure period, **(e)** during the road closure period, and **(f)** after the traffic resumed period.

https://doi.org/10.1371/journal.pone.0321970.g007

Dataset B is the only datasets among these four that showed a clear decrease in trip volumes for closed road segments during the closure period (Fig. 8b). For trips that were from west to east, the number of trips dropped from between 60–90 trips per day prior to the road closure to less than 10 trips per day from June 11th to June 15th (closure period), and nearly no trips for the period from June 16th to 22nd. For the trips that were from east to west, the number dropped from 50 to 60 trips per day to nearly no trips during the road closure period. For trips that followed the local detour routes, the numbers increased from less than 5 trips per day before the road closure period to around 15–20 trips per day during the closure.

For dataset C, the number of trips that went through the road closure didn't show a clear change of pattern for the road closure period compared with before the road closure (Fig. 8c). Dataset D showed a slight increase in trips that traveled through the closed road segments for the road closure period compared with before the road closure (Fig. 8d). The number of trips that followed the detours showed a slight increase, from 10 trips per day to around 20 and 30 trips for datasets C and D respectively.

For the period when traffic had resumed, dataset B showed a clear "back to normal" pattern from June 22nd to 24th and showed a very similar number of trips for all routes from the 24th to 29th compared with the period before the road closure (Fig. 8b). For datasets A and C, the patterns for trips during the period when traffic had resumed were similar to those during the road closure period. Dataset D showed an increase in trip numbers for all three routes from June 25th on (Fig. 8d).

Based on these findings, datasets A, C, and D were suspect from the perspective of trip-level analysis.

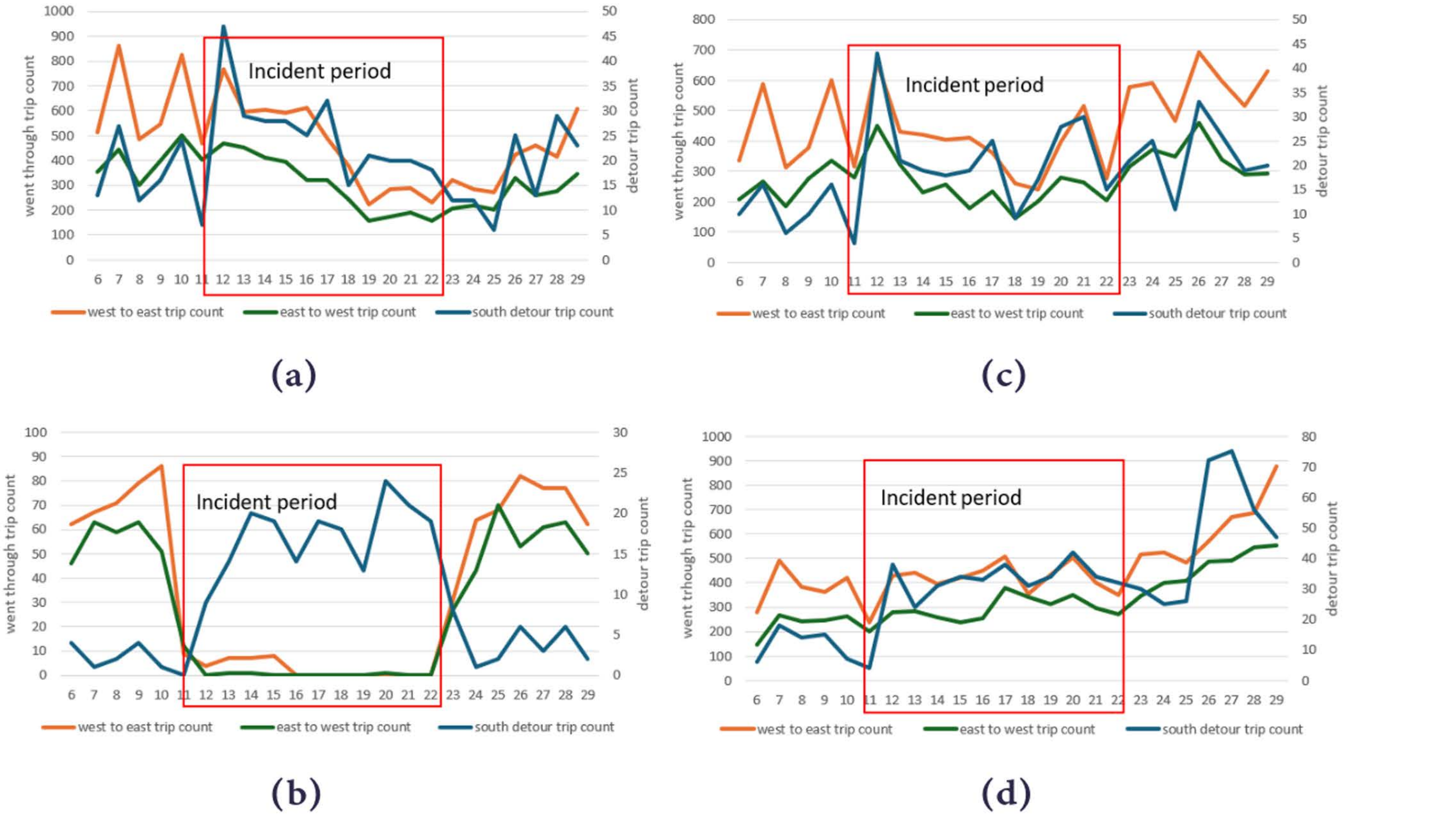

Fig 8. Number of trips that went through the closed road segments on I-95 and the local detour along different routes we designed for (a) dataset A, (b) dataset B, (c) dataset C, and (d) dataset D. The number of trips that travel through the detour is shown on the second axis since there are significantly fewer trips in this category.

https://doi.org/10.1371/journal.pone.0321970.g008

Case 2, Los Angeles, CA, I-10 closure

The number of devices extracted in the study area from the 5 datasets for case 2 showed 2 types of patterns (Fig. 9a). Datasets B and E showed a similar pattern with an average of around 2000 devices over most of the days for the three time periods and showed a decrease from the 26th November on. The device numbers for datasets A, C, and D showed more severe fluctuations in the device numbers over the days, and these fluctuations were very similar across each of the datasets. For the period before the road closure, datasets A, C, and D showed a peak in device numbers on the 6th or 7th of November and then fell back to normal levels. For the period during the road closure, the device numbers increased from November 11th to 13th for all 3 datasets and then decreased from the 13th to 16th. For the period after traffic had resumed, the number of devices from dataset D increased from the 19th to 25th, while the number of devices for datasets C and D didn't change significantly.

The number of waypoints (Fig. 9b) from the datasets were similar to the patterns returned by the analysis of the numbers of devices. Patterns for dataset B and E were also similar as the pattern of the devices number over the days except for the fluctuation happened since 23rd. Prior to the road closure, datasets A and C didn't change much, while dataset D showed a peak in the number of waypoints on the 5th and 6th. During the road closure period, datasets A and C showed peaks on the 14th while dataset D showed a larger fluctuation with two peaks in the number of waypoints on the 13th and 18th. After the road closure, the number of waypoints was generally stable, while dataset D showed an increase from the 21st with another drop on the 26th.

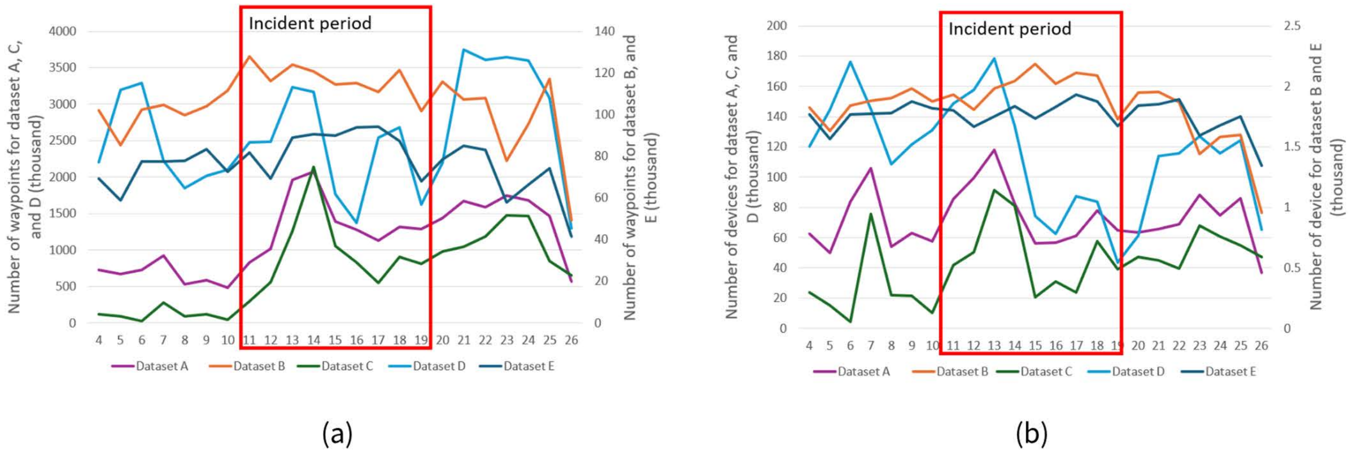

Fig 9. (a) the number device for different datasets over the study time period, due to datasets A and B containing significantly fewer waypoints and devices, we used the second axis to represent datasets A and B.

https://doi.org/10.1371/journal.pone.0321970.g009

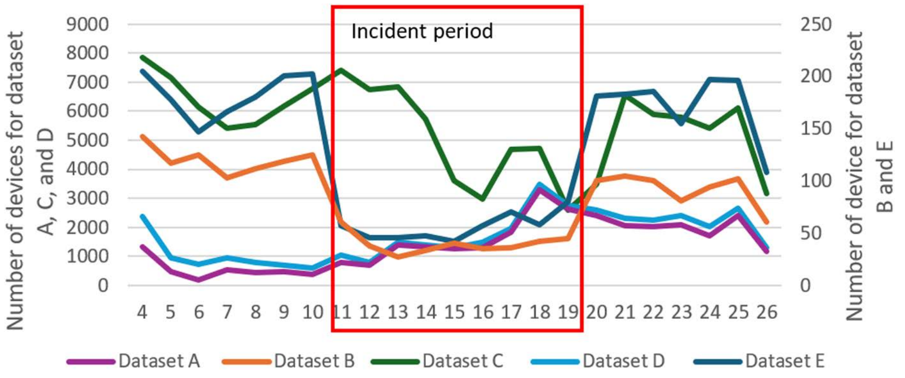

Fig 10. Number of devices that were matched to closed road segments on the I-10 for the study period. Number of devices for datasets B and E is shown along the second axis. The road closure period was bounded in red.

https://doi.org/10.1371/journal.pone.0321970.g010

Devices that were matched to the road closure in case 2

We then investigated the number of devices that were matched to the closed road segments after the map matching. For before the road closure period, the number of devices for datasets A and C didn't change much and the number showed a slight decrease from the 4th to the 7th before bouncing back from the 7th to the 10th (Fig. 10).

During the road closure, datasets B and E showed a significant drop in the number of devices that were matched to the road segments, decreasing from an average of around 100 and 150 devices per day respectively to around 50 devices per day from November 11th, the first day of the road closure. Dataset D showed a slight decrease from the 13th but also showed a peak on the 17th and 18th. Datasets A and C showed a general increase in the number of devices during the road closure.

After the road was reopened and traffic had resumed, the number of devices from datasets D, and E bounced back to the levels seen before the incident. Dataset B also showed the bounce-back pattern, but the device number from dataset

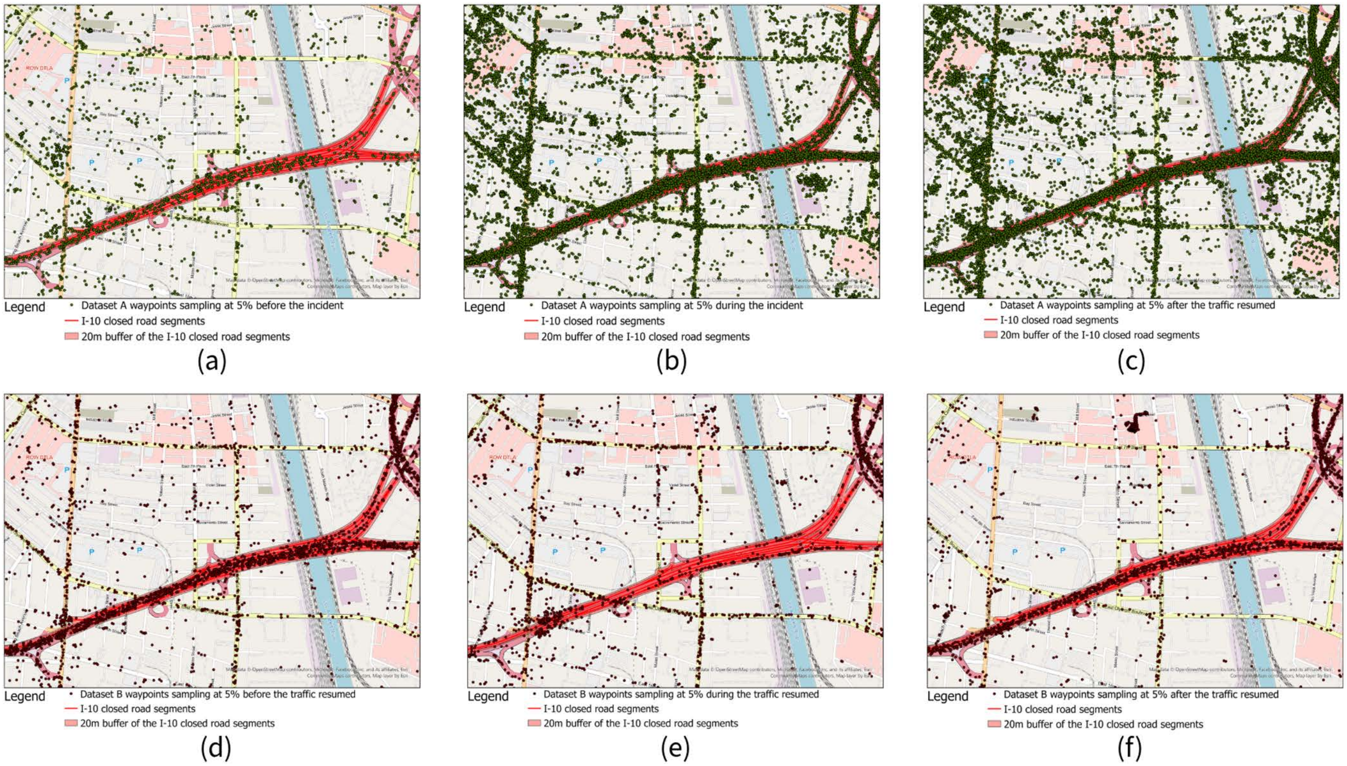

Fig 11. Waypoints randomly sampled at 5% for dataset A in the I-10 road closure area for (a) before the road closure period, (b) during the road closure period, and (c) after the traffic resumed period. And waypoints randomly sampled at 5% for dataset B in study area for **(d)** before the road closure period, **(e)** during the road closure period, and **(f)** after the traffic resumed period.

https://doi.org/10.1371/journal.pone.0321970.g011

B for after the traffic had resumed was slightly less than before the incident. The number of devices from datasets A and C didn't change much during this period.

Mapping the waypoints from the different datasets over the different time periods reflected our findings from the map-matching results. For dataset A, compared with the period before the incident (Fig. 11a), even more waypoints showed in the closed road segments during the road closure period (Fig. 11b). Waypoints showed that for the period after the traffic resumed were slightly less than during the road closure period (Fig. 11c). On the contrary, dataset B showed an expected travel pattern where compared with before the road closure (Fig. 11d) the number of waypoints decreased significantly during the road closure (Fig. 11e). After the traffic resumed, the number of waypoints on closed road segments showed a clear bounce back (Fig. 11f). However, we could still see that some of the waypoints occurred in the road closure area, as shown in the plot of the number of devices matched to the closed segments (Fig. 10). Datasets B and E showed a similar pattern for the number of waypoints on the map. Datasets C and D showed a similar pattern to dataset A, with more waypoints being present during the road closure compared with before the road closure.

Trip-level analysis for case 2

For trip-level analysis of the 5 datasets, since all 4 routes reflected trips that went through the road closure and no detour trips, we found that the trip numbers for the 4 routes in each dataset showed a similar pattern. Before the road closure, datasets A, C, and D showed few fluctuations in trips over these days before the closure (Fig.12a, c, and d) with datasets A and C showing fewer trips for all 4 routes (Fig.12a and c). Dataset B had a peak in the number of trips on November

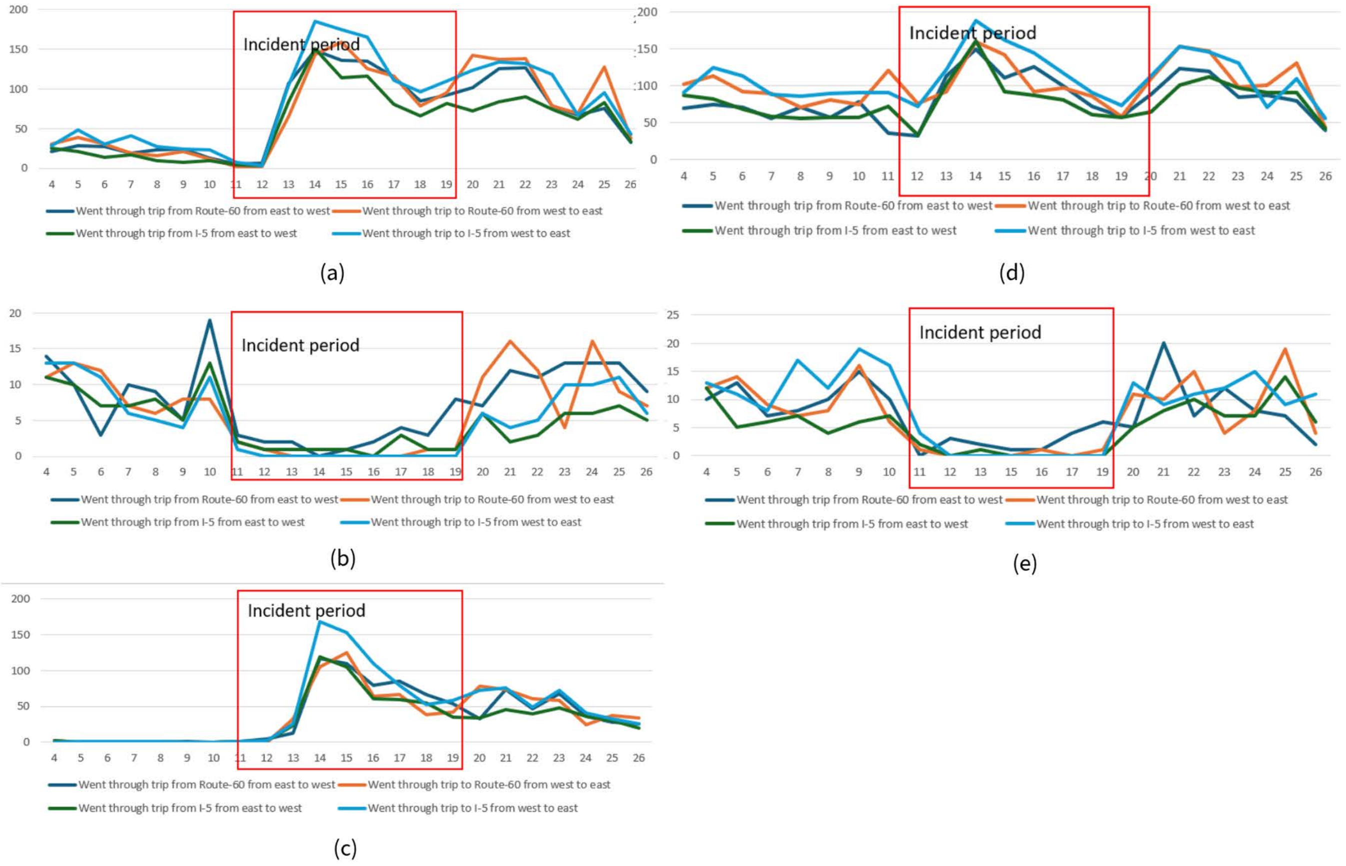

Fig 12. Number of trips on closed road segments on I-10 along the 4 different routes for (a) dataset A, (b) dataset B, (c) dataset C, (d) dataset D, and (e) dataset E.

https://doi.org/10.1371/journal.pone.0321970.g012

10th (Fig. 12b). Dataset E had a peak on the 9th for trips of two directions via SR-60 and trips to I-5 from west to east and another peak on the 7th for the trips going to the I-5 from west to east (Fig. 12e).

During the road closure, datasets B and E showed a clear decrease in the number of trips for all 4 routes on the 11th and the number of trips was nearly zero for the entire road closure period from the 11th to 19th (Fig.12b and e). However, datasets A, C, and D showed an unexplained increase in the number of trips during the road closure period. The number of trips for these three datasets showed a significant increase (from an average of around 20 to more than 140 trips for dataset A, from nearly 0 to more than 120 trips for dataset C, and from an average of around 80 to more than 140 trips for dataset D) on the 13th and 14th when it was expected that the number of trips would be decreased. The number of trips from these three datasets showed a pattern of decrease from the 14th to the 18th, but the trip numbers were higher than before the road closure for datasets A and C.

After traffic had resumed (e.g., from the 20th), the number of trips from datasets B and E bounced back to before closure levels. The number of trips from datasets A and D showed an increase on the 19th and 20th. Dataset C showed some degree of increase in the number of trips for the two routes that went through SR-60, but not much of an increase for the two routes that went through the I-5.

With information about the road closure and based on this analysis, datasets A, C, and D were suspect of having errors or biases.

Discussion

With the growing adoption of mobile devices, passively collected LBS data has become more available and brings opportunities for analysis of travel behaviors on a large scale and at fine granularity [1,2]. Despite the new advantages that LBS data offers, data quality assessment and preprocessing have always been indispensable steps for researchers who desire to have reliable analysis results [2,15]. Multi-dimensional data assessments have been discussed in previous research, such as dimensions of accuracy and consistency [8]. In this research, we discovered and examined trip-level errors in 2023 LBS data that violated spatial and temporal consistency by showing trips at a time and place where there shouldn't be any trips. This recently emerged data quality issue hasn't been discussed or examined by previous studies to the best of our knowledge and could otherwise be neglected. The trip-level errors that we discovered would likely have a significant impact on analyses using LBS data, for example, travel behavior analysis would be error-prone if it was based on trip-level error, and traffic monitoring results would be highly biased if the dataset used contains a certain proportion of trip-level errors. Therefore, we believe that LBS data users from all fields should consider including a trip-level error examination prior to undertaking their analyses.

Based on the contrapositive of the understanding that there should be no trips that go through a closed road during a road closure, we formed the assumption that the data quality of an LBS dataset is suspect if it contains trips that show travel through closed road segments during a road closure. To quantify the trip-level error, we designed a trip-level error examination workflow based on scenarios that involve road closures. The workflow didn't only focus on waypoints or devices that had been matched to the road closure but also examined the number of trips that went through the closed road segments following the designed routes (routes that went through the closed segments and local detours). We compared the number of trips that went through the road closures during the time of the closure with the number of trips during other time periods (e.g., before and after the closure). Although the workflow was designed based on scenarios that involved road closures, we believe datasets that contain trip-level errors are overall suspect with respect to data quality and may also contain other trip-level errors.

In this research, we selected two real-world cases involving road closure caused by severe incidents that happened in Philadelphia, PA, and Los Angeles, CA, respectively for trip-level error examination. We examined 4 and 5 LBS datasets for the two cases respectively acquired from data vendors in the US. After the trip-level examination, we found 3 datasets in both cases contained significant number of trip-level error and therefore suspect. We have communicated with all of the data vendors we used about our findings on the trip-level errors, and some of the data vendors are indicating that they are now undertaking more careful scrutiny of the data and putting in place their own data quality procedures to ensure a higher quality of data. However, a trip-level examination will still be necessary even if all data vendors could provide data with high quality and without any trip-level errors since datasets that have already been on the market or acquired by data users may still contain trip-level errors.

This study lays the groundwork for further exploration of trip-level errors in LBS data. As for the limitation of this research, in order to avoid introducing biases into the trip-level analysis (e.g., excluding part of the waypoints or trips in data preprocessing), we used raw data in the trip-level analysis. Waypoint-level data noise may have a potential impact on the map-matching results (e.g., some waypoints might be matched to the nearby road segment due to data noise), but the number of trips that went through will not be impact since the trip-level analysis consider the number of trips went through the designed routes instead of just the number of waypoints that were matched to the closed road segments. The location based service data may also bring bias to our data analysis results since the data may not be evenly sampled across all regions and/or among all populations.

In addition, although we examined trip-level errors based on accident scenarios that involved road closures, we believe road closures will not be the only scenarios for trip-level data quality checks. Other scenarios could also be used for trip-level quality checks, such as different commuting patterns between holidays and weekdays, travel involving moving bridges or features that may block traffic periodically. We will further investigate workflows that distinguish valid trips from

erroneous ones and investigate the potential reasons of the trip-level error in future research. This research further highlights the importance of data preprocessing and cleaning, and running consistency checks on LBS datasets being used for analysis.

Author contributions

Conceptualization: Peiqi Zhang, Kathleen Stewart, Aref Darzi.

Data curation: Aref Darzi.

Formal analysis: Peiqi Zhang, Aref Darzi.

Funding acquisition: Kathleen Stewart, Aref Darzi.

Methodology: Peiqi Zhang, Kathleen Stewart, Aref Darzi.

Resources: Aref Darzi.

Validation: Peiqi Zhang.

Visualization: Peiqi Zhang.

Writing – original draft: Peiqi Zhang, Kathleen Stewart.

Writing – review & editing: Peiqi Zhang, Kathleen Stewart.

References

1. Wang F, Chen C. On data processing required to derive mobility patterns from passively-generated mobile phone data. Transportation Research Part C: Emerging Technologies. 2018;87(1):58–74.
2. Xu Y, Li X, Shaw SL, Lu F, Yin L, Chen BY. Effects of data preprocessing methods on addressing location uncertainty in mobile signaling data. Annals of the American Association of Geographers. 2020;111(2):515–39.
3. Chen C, Ma J, Susilo Y, Liu Y, Wang M. The promises of big data and small data for travel behavior (aka human mobility) analysis. Transp Res Part C Emerg Technol. 2016;68:285–99. https://doi.org/10.1016/j.trc.2016.04.005 PMID: 27182125
4. Gadziński J. Perspectives of the use of smartphones in travel behaviour studies: Findings from a literature review and a pilot study. Transportation Research Part C: Emerging Technologies. 2018;88:74–86.
5. Cheng L, Cai X, Liu Z, Huang Z, Chen W, Witlox F. Characterising travel behaviour patterns of transport hub station area users using mobile phone data. Journal of Transport Geography. 2024;116(1).
6. Yu Q, Li W, Yang D, Zhang H. Mobile phone data in urban commuting: A network community detection-based framework to unveil the spatial structure of commuting demand. J Advanced Transportation. n.d.;2020.
7. Baird T, Stinger P, Cole E, Collins R. Mobile device data for parks and public lands transportation planning: A framework for evaluation and applications. Transportation Research Record. n.d.:490–500.
8. Pan Y, Darzi A, Yang M, Sun Q, Kabiri A, Zhao G. National-level multimodal origin–destination estimation based on passively collected location data and machine learning methods. Transportation Research Record. 2023;2023(1).
9. Grantz KH, Meredith HR, Cummings DAT, Metcalf CJE, Grenfell BT, Giles JR. The use of mobile phone data to inform analysis of COVID-19 pandemic epidemiology. Nature Communications. 2020;11(1):1–10.
10. Gao S, Rao J, Kang Y, Liang Y, Kruse J. Mapping county-level mobility pattern changes in the United States in response to COVID-19. SIGSpatial Special. 2020;12(1):16–26.
11. Zhang L, Darzi A, Ghader S, Pack ML, Xiong C, Yang M, et al. Interactive COVID-19 Mobility Impact and Social Distancing Analysis Platform. In: Transportation Research Record. SAGE Publications Ltd; 2023. p. 168–80.
12. Merry K, Bettinger P. Smartphone GPS accuracy study in an urban environment. PLoS One. 2019;14(7).
13. Wang S, Bao Z, Culpepper JS, Cong G. A Survey on Trajectory Data Management, Analytics, and Learning. Vol. 54, ACM Computing Surveys. Association for Computing Machinery; 2021.
14. Buchin M, Driemel A, van Kreveld M, Sacristán V. Segmenting trajectories: A framework and algorithms using spatiotemporal criteria. Journal of Spatial Information Science. 2011;3(2011):33–63.
15. Ferreira E, Ferreira D. Towards altruistic data quality assessment for mobile sensing. UbiComp/ISWC 2017 - Adjunct Proceedings of the 2017 ACM International Joint Conference on Pervasive and Ubiquitous Computing and Proceedings of the 2017 ACM International Symposium on Wearable Computers. n.d.:464–9.

16. Järv O, Tenkanen H, Toivonen T. Enhancing spatial accuracy of mobile phone data using multi-temporal dasymetric interpolation. International Journal of Geographical Information Science. 2017;31(8):1630–51.
17. Zhang P, Stewart K, Li Y. Estimating traffic speed and speeding using passively collected big mobility data and a distributed computing framework. Transactions in GIS. 2023;27(4):1124–44.
18. Deak MD, Porter WP, Mathewson PD, Lovelace DM, Flores RJ, Tripati AK, et al. Metabolic skinflint or spendthrift? Insights into ground sloth integument and thermophysiology revealed by biophysical modeling and clumped isotope paleothermometry. J Mamm Evol. 2025;32(1):1. https://doi.org/10.1007/s10914-024-09743-2 PMID: 39822851
19. Olama M, Djouadi S, Papageorgiou I, Charalambous C. Position and velocity tracking in mobile networks using particle and Kalman filtering with comparison. IEEE Transactions on Vehicular Technology. 2008;57(2):1001–10.
20. Dyrmishi S, Hadachi A. Mobile positioning and trajectory reconstruction based on mobile phone network data: A tentative using particle filter. Proceedings of the 2021 7th International Conference on Models and Technologies for Intelligent Transportation Systems (MT-ITS 2021). 2021:N/A. https://doi.org/N/A
21. Zheng Y. Trajectory data mining: An overview. ACM Transactions on Intelligent Systems and Technology. 2015;6(3):1–41.
22. Pereira FM, Sofia RC. An Analysis of ML-Based Outlier Detection from Mobile Phone Trajectories. Future Internet. 2023 Jan 1;15(1).
23. Zaharia M, Xin RS, Wendell P, Das T, Armbrust M, Dave A, et al. Apache spark: A unified engine for big data processing. Communications of the ACM. 2016;59(11):56–65.
24. Yu J, Zhang Z, Sarwat M. Spatial data management in apache spark: the GeoSpark perspective and beyond. Geoinformatica. 2019;23(1):37–78.
25. Mathew S, Varia J. Overview of amazon web services. Amazon Whitepapers. n.d.;105(1):22.
26. OpenStreetMap contributors. Road network retrieved from OpenStreetMap. 2023.
27. Zaharia M, Chowdhury M, Franklin M, Shenker S, Stoica I. Spark: Cluster computing with working sets. Proceedings of the 2nd USENIX Workshop on Hot Topics in Cloud Computing (HotCloud 10). n.d.
28. Wu Z, Xie J, Wang Y, Nie Y, . Map matching based on multi-layer road index. Transportation Research Part C: Emerging Technologies. 2020;118.
29. Quddus M. High integrity map matching algorithms for advanced transport telematics applications. 2006.
30. Fan J, Fu C, Stewart K, Zhang L. Using big GPS trajectory data analytics for vehicle miles traveled estimation. Transportation Research Part C: Emerging Technologies. 2019;103(March):298–307. https://doi.org/10.1016/j.trc.2019.04.019
